# Climate Impacts From a Removal of Anthropogenic Aerosol Emissions

**DOI:** 10.1002/2017GL076079

**Published:** 2018-01-08

**Authors:** B. H. Samset, M. Sand, C. J. Smith, S. E. Bauer, P. M. Forster, J. S. Fuglestvedt, S. Osprey, C.-F. Schleussner

**Affiliations:** 1 CICERO Center for International Climate and Environmental Research, Oslo, Norway,; 2 School of Earth and Environment, University of Leeds, Leeds, UK,; 3 NASA Goddard Institute for Space Studies and Columbia Earth Institute, New York, NY, USA,; 4 National Centre for Atmospheric Science and Department of Physics, University of Oxford, Oxford, UK,; 5 Climate Analytics, Berlin, Germany

## Abstract

Limiting global warming to 1.5 or 2.0°C requires strong mitigation of anthropogenic greenhouse gas (GHG) emissions. Concurrently, emissions of anthropogenic aerosols will decline, due to coemission with GHG, and measures to improve air quality. However, the combined climate effect of GHG and aerosol emissions over the industrial era is poorly constrained. Here we show the climate impacts from removing present-day anthropogenic aerosol emissions and compare them to the impacts from moderate GHG-dominated global warming. Removing aerosols induces a global mean surface heating of 0.5–1.1°C, and precipitation increase of 2.0–4.6%. Extreme weather indices also increase. We find a higher sensitivity of extreme events to aerosol reductions, per degree of surface warming, in particular over the major aerosol emission regions. Under near-term warming, we find that regional climate change will depend strongly on the balance between aerosol and GHG forcing.

## Plain Language Summary

To keep within 1.5 or 2° of global warming, we need massive reductions of greenhouse gas emissions. At the same time, aerosol emissions will be strongly reduced. We show how cleaning up aerosols, predominantly sulfate, may add an additional half a degree of global warming, with impacts that strengthen those from greenhouse gas warming. The northern hemisphere is found to be more sensitive to aerosol removal than greenhouse gas warming, because of where the aerosols are emitted today. This means that it does not only matter whether or not we reach international climate targets. It also matters how we get there.

## 1. Introduction

If global warming is to be kept within 1.5 or 2.0°C, strong, and rapid mitigation of anthropogenic greenhouse gas (GHG) emissions is required (Matthews & Caldeira, 2008; Millar et al., 2017; Rogelj, Luderer, et al., 2015). As anthropogenic aerosols are often coemitted with long-lived GHG, such emissions will likely also see sharp decreases—compounded by present and future effort to improve air quality (Bowerman et al., 2013; Smith & Bond, 2014). The combined climate effect of GHG and aerosol emissions over the industrial era is, however, poorly constrained (Bindoff et al., 2013; Boucher et al., 2013; Myhre, Shindell, et al., 2013). Predicting the net impact of a removal of anthropogenic aerosols is therefore challenging, but nevertheless critically important due to the difference in regional patterns between present GHG and aerosol forcing (Myhre et al., 2017). It is a reasonable expectation that the strongest radiative forcing will occur near emission regions, which are also among the main populated regions of the globe. This indicates that the spatial details of emissions scenarios must be considered, rather than just the total radiative forcing and realized global warming (Wang et al., 2017).

As the most notable drivers of radiative forcing (RF) (Myhre, Shindell, et al., 2013), the separate impacts of GHG and aerosol changes are well studied, but mainly through idealized step perturbations (e.g., Andrews et al., 2010; Kvalevåg et al., 2013; Samset et al., 2016). However, their relative contributions to historical and near-term climate change, in terms of global and regional changes to climate indicators such as temperature, precipitation, and extreme weather, are less understood. For instance, emission pathways such as Representative Concentration Pathway (RCP) 2.6 also rapidly reduce aerosol emissions over the 21st century (Rogelj, Malte, et al., 2015), allowing information only on the combined impact of both changes. The present poor constraints on aerosol RF (Myhre, Shindell, et al., 2013; Samset et al., 2014) add considerable uncertainty to the relative contributions of GHG and aerosol emission reductions to future temperature, precipitation, and extreme weather change.

In the present study, we use idealized scenarios simulated by four global, fully coupled atmosphere-ocean-composition climate models to separate the climatic effects of aerosol emission mitigation from those of continued, moderate greenhouse gas-induced warming. In the following, we show the simulated responses to a removal of anthropogenic emissions of sulfate and carbonaceous aerosols (black and organic carbon), compared to moderate (1.2–1.7°C) GHG-dominated warming, in terms of global and regional temperatures, precipitation, and extreme weather indices. We also investigate the sensitivity of key climate variables to the two types of forcing.

## 2. Methods

The models participating in the present study are Community Earth System Model version 1 Community Atmospheric Model version 5 (CESM1 CAM5) (Hurrell et al., 2013), Goddard Institute for Space Studies E2-R One-Moment Aerosol (GISS-E2-R OMA) (Koch et al., 2011; Schmidt et al., 2014), Norwegian Earth System Model 1 - medium resolution (NorESM1) (Bentsen et al., 2013; Iversen et al., 2013), and Hadley Centre Global Environmental Model, version 2 Carbon Cycle Stratosphere (HadGEM2-CCS) (Collins et al., 2011; Martin et al., 2011). All models were set up with fully coupled oceans. Three simulations were performed by each model:(i) a preindustrial baseline, representative of year 1850; (ii) CO_2_ concentration abruptly changed to achieve a global, annual mean warming of around 1.5°C relative to case (i)—all other climate forcers, including aerosol emissions, were kept at present-day conditions; and (iii) identical to (ii) except that anthropogenic emissions of SO_2_, and fossil fuel black carbon and organic carbon, were set to 0. Natural sources and nitrate aerosol emissions were set to present-day levels. For each case, 100 years were run after the perturbation, which has been shown to be sufficient in the present generation of models to equilibrate all but the centennial ocean heat uptake (Caldeira & Myhrvold, 2013; Samset et al., 2016). Figure S1 in the supporting information shows that for the present analysis, the model responses have insignificant trends over the time periods used. The analysis was performed over the last 50 simulated years, except for HadGEM2-CCS where only years 71–100 were available.

For cases (ii) and (iii), models CESM1 CAM5, GISS-E2-R OMA, NorESM1, and HadGEM2-CCS ran with CO_2_ concentrations of, respectively, 420, 406, 430, and 420 ppm. These values were set to achieve an approximate warming of 1.5°C, taking into account the varying climate sensitivities of the models. In the end, the models simulated a year 51–100 warming of 1.7, 1.4, 1.2, and 1.1°C relative to case (i). For geographical multimodel comparisons, all final model output were converted to a common resolution of 2.5° × 1.9° using first-order conservative remapping (Jones, 1999). Aerosol emission inventories were used in Coupled Model Intercomparison Project Phase 5 (CMIP5) (Lamarque et al., 2010).

GISS-E2-R OMA was run with interactive gas phase chemistry scheme and includes treatment of the direct, the first indirect, and semidirect (rapid adjustment) aerosol radiative effects. Snow albedo feedback from black carbon (BC) emissions were also included. Ozone and nonprescribed aerosols were allowed to react freely.

CESM1 CAM5 was run with a bulk aerosol module, but with full direct, indirect, and semidirect (rapid adjustment) aerosol radiative effects included. Effects of black carbon on snow were not included. Simple gas-phase chemistry was included for sulfate aerosol. Ozone concentrations were prescribed at present-day levels.

HadGEM2-CCS contains the Coupled Large-scale Aerosol Simulator for Studies In Climate aerosol scheme (Bellouin et al., 2011). Full direct, indirect, and semidirect (rapid adjustment) aerosol radiative effects were included. HadGEM2-CCS does not include tropospheric chemistry but does include SO_2_ and DMS oxidation to SO_4_ based on climatological concentrations of OH, HO_2_, H_2_O_2_, and O_3_. Effects of black carbon on snow were not included.

NorESM1, based on National Center for Atmospheric Research CAM4 extended with an aerosol-cloud-radiation scheme, includes full direct, indirect, and semidirect (rapid adjustment) aerosol radiative effects. The aerosol lifecycle scheme calculates mass concentrations of sea salt, mineral dust, particulate sulfur, BC, and primary and secondary organics tagged to production mechanism. Ozone concentrations are prescribed. Albedo feedback from BC deposition on snow were also included.

To identify differences in regional response patterns between GHG-induced warming and that from aerosol emission reductions, we define the aerosol sensitivity ratio:

$${\text{ASR}}_{X}=|{\left(\Delta X/\;<\;\Delta T\;>\right)}^{\text{aero}}/{\left(\Delta X/\;<\;\Delta T\;>\right)}^{\text{GHG}}|$$

where *X* is a climate variable (temperature, precipitation, or extreme weather index, as function of latitude and longitude), GHG refers to the GHG-dominated simulation, and aero refers to the impacts of removing anthropogenic aerosols. The absolute value is taken to highlight the difference in sensitivity rather than sign.

Following recent literature (Schleussner et al., 2016; Sillmann, Kharin, Zhang, et al., 2013), we calculate three extreme weather indices: TXx, intensity of hot extremes, defined as the annual maximum value of daily maximum temperature; CDD, consecutive dry days, defined as the annual maximum number of consecutive days for which the precipitation is below 1 mm per day; and RX5D, heavy precipitation intensity, defined as the annual maximum precipitation for a consecutive 5 day period. (See section 4 for a note on the ability of present models to simulate such changes.)

For the temperature and precipitation changes relative to case (i), the statistical significance of changes relative to the preindustrial control was tested at each grid point using a two-tailed Student’s *t* test, with degrees of freedom equal to the number of simulated years in all models (4 * 50 = 200). To take into account multiple p-testing, the traditional requirement of *p* < 0.05 was strengthened to *p* < 0.02, following the analysis and methods presented in recent literature (Ventura et al., 2004). For the extreme indices, we make the simplified test of checking whether all models calculate a signal of the same sign.

To study regions of high anthropogenic aerosol emissions, we define the following region boxes: Europe (0–45°E,40–60°N), U.S. (125–75°W,30–50°N], and East Asia (75–135°E, 15–45°N].

**Figure 1.**
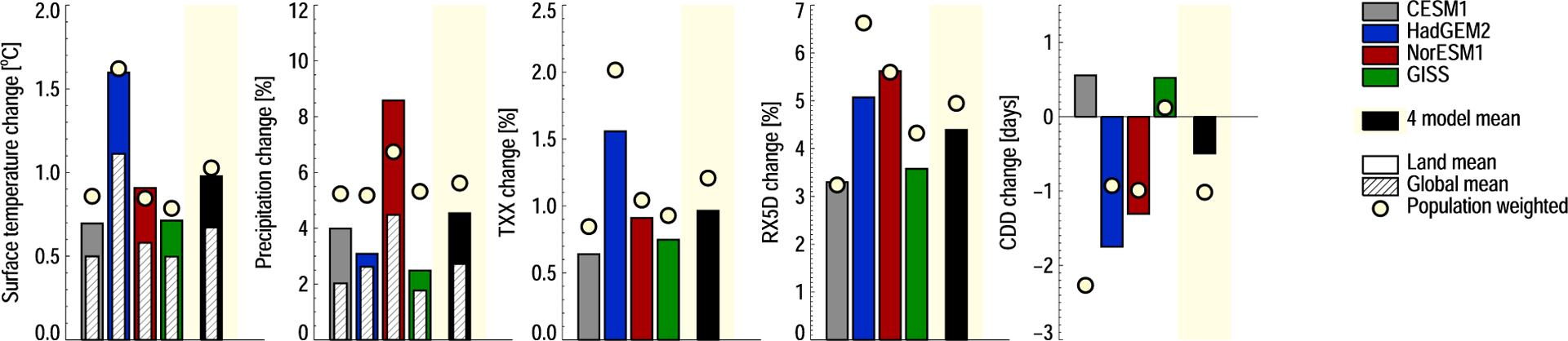
Model responses to a removal of anthropogenic aerosol emissions. (left to right) Changes to surface temperature, precipitation, TXx, RX5D, and CDD. The solid bars show land area means; the hatched bars show global means. The yellow circles show changes over land, weighted by population density. The black bars show the multimodel mean responses.

For the regional analysis (Figure 4), only grid points with land fractions >50% were selected, and the results were weighted by the land fraction and the area of the grid box. To weigh by present population density (Figure 1), we used the GPWv3 Population Density Grid for year 2000, available at http://sedac.ciesin.columbia.edu/gpw, converted to the multimodel resolution as described above. No extrapolation for future population growth was made.

## 3. Results

In this section, we first discuss the modeled response to a total removal of anthropogenic sulfate and carbonaceous aerosols. We then compare the geographical sensitivity patterns to GHG-dominated warming and aerosol reductions. Absolute model responses are shown in Table 1, and the geographical distributions from individual models are shown in Figures S1–S4.

Figure 1 summarizes the climate and extreme event responses to a removal of anthropogenic aerosols, from a world with around 1.5°C GHG-dominated warming. Global surface temperature is predicted to increase by0.7°C (multimodel mean, model range is 0.5–1.1°C), while the land surface warms by 1.0°C (model range0.7–1.6). As sulfate is the dominant aerosol surface temperature driver for present-day emissions (Baker et al., 2015), this large intermodel spread is likely driven by differences in modeled response to SO_2_ emission changes (Baker et al., 2015; Kasoar et al., 2016; Samset et al., 2016). Recently discussed factors include the dynamical atmospheric response to a strongly regional perturbation, differences in parameterizations of aerosol-cloud interactions, and the cloud fields of the host model. However, no study has yet pointed to a single, dominant cause for intermodel differences in surface temperature response to changes in SO_2_ emissions. Broadly, differences in modeled climate response to aerosol perturbations are also known to be affected by transport processes, wet removal, and aging. The present study uses model versions and emission estimates similar to those compared in AeroCom Phase II (Myhre, Samset, et al., 2013; Samset et al., 2013), with modeled burden differences when removing anthropogenic aerosol emissions consistent with those studies.

We note that in two models, Arctic warming due to aerosol reductions reaches 4°C in some locations (Figures S2–S5). The four-model mean increase for the 60°N–90°N region is 2.8°C. Global mean precipitation increases by 2.8% (7.5% for the 60°N–90°N region). For the extreme weather indices, where we calculate means for the land surface only, mean maximum daily temperature (TXx) increases by 0.9 (0.6–1.5) °C, and maximum 5 day precipitation (RX5D) by 4.1 (3.1–5.3)%. The modeled change in consecutive dry days (CDDs) varies in sign, with a multimodel mean of −0.5 days, but a range of −1.7 to 0.5.

Overall, we find an increase in both temperature, precipitation, and extreme weather as a result of removing anthropogenic aerosols. Hence, the dominating effect of removing aerosols is the loss of present-day sulfate-induced cooling. The broad response patterns are consistent with those from moderate GHG-dominated warming, in both our own simulations (Table 1), and previous studies of the impacts of a 1.5°C warming (e.g., Schleussner et al., 2016; Wang et al., 2017). Globally, the effect of aerosol removal is therefore a strengthening of the climate impacts already seen and expected for the near future.

Next, we move beyond global means to study the regional distribution of impacts. As aerosols have short atmospheric residence times, their RF is strongest near emission sources—which are in turn concentrated near populated areas. Impacts may, however, have a widespread distribution, due to teleconnections and circulation changes. In Figure 1, the yellow circles show the land area means when weighted by population density. For most changes, these changes are markedly stronger than the mean for all land, with good agreement between models (again, with the exception of CDD). For example, TXx change is on average 25% stronger in populated areas. Regardless of whether current global models are able to simulate realistic changes in climate response and extremes, this result is a reminder that the total land area response may not be representative of changes in populated regions (Frame et al., 2017).

Whichever emission pathway is taken toward 2100, the mix of GHG and aerosol radiative forcing can be expected to change with time. Differences in sensitivity of impacts to warming from GHG and aerosol-driven warming can therefore cause regional climate change, even in a case where global warming stays close to 1.5 or 2°C for a longer time.

Recent literature has discussed the sensitivity of the climate to aerosol emission changes, relative to changes in GHG concentration. Most studies have, however, investigated increases in aerosol emissions, which may have different impacts to a removal. Our results indicate an apparent hydrological sensitivity (AHS, the total precipitation response per degree of surface warming (Fläschner et al., 2016)) to aerosol reduction of around 4%. This is in line with previous studies (Schleussner et al., 2016). We also find an AHS for GHG increase of 4%, which is higher than in some other studies, but consistent within our model spread (1.8%–7.5%).

**Figure 2.**
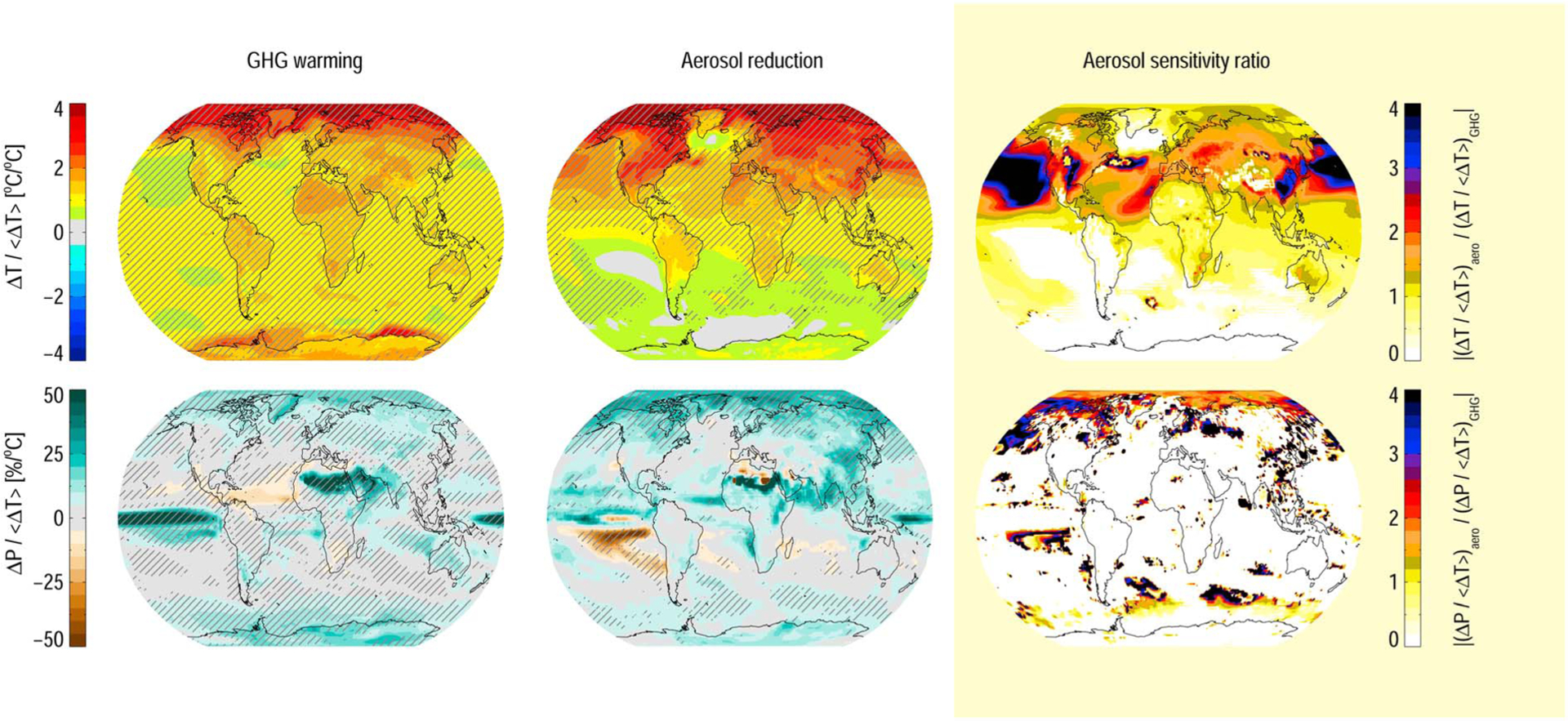
Regional sensitivities to increases in (left column) long-lived GHG concentrations and (middle column) aerosol emission removal. (top row) Temperature. (bottom row) Precipitation. All panels show the mean of four models. (right column) The aerosol sensitivity ratio (ASR; see section 2). The hatched regions are where the multimodel mean is significantly different from the baseline mean, according to a two-tailed Student’s *t* test with *p* < 0.02 (see section 2). ASR is only plotted where both the GHG and aerosol change are statistically significant.

Since the aerosol distribution is more heterogeneous compared to well-mixed GHG, the rest of our discussions will focus on the regional sensitivities to GHG increase relative to aerosol removal. Figure 2 shows the multimodel geographical responses to GHG increase (Figure 2, left column), and aerosol emission removal (Figure 2, middle column), normalized by the global mean surface temperature change in each model. For temperature change (Figure 2, top row), this shows the relative warming of different parts of the world. Following well-known patterns (Collins et al., 2013; Joshi et al., 2008; Screen & Simmonds, 2010; Sutton et al., 2007), the models predict a warming that is strongest in the polar regions, and stronger warming over land than over oceans. This pattern is similar for aerosols, however, with even stronger polar warming, and hot spots around the major anthropogenic aerosol emission regions (mainly China and the U.S.). To compare the two, we construct the aerosol sensitivity ratio (ASR; see section 2) in Figure 2 (right column). Here we clearly see an elevated sensitivity to aerosol reductions in the northern hemisphere, relative to GHG increase. We attribute this mainly to the hemispherical asymmetry of present-day aerosol emissions. Note that the ASR is plotted only for model bins where both the GHG and aerosol-induced changes are significantly different from 0 (see section 2). We also find an area south of Greenland with weak or no ocean surface warming when reducing aerosol emissions, visible in all participating models (see Figures S2–S5). This resembles the expected pattern from a slowdown of the Atlantic Meridional Overturning Circulation (AMOC) (Rahmstorf et al., 2015). We do not follow up on this point here but link it to the anomalous northern hemispheric warming introduced by aerosol reductions that has previously been shown to lead to a reversible AMOC slowdown on multidecadal time scales (Levermann & Meinshausen, 2014).

The apparent hydrological sensitivities, i.e., including both rapid adjustments and the response to surface temperature changes (Figure 2, bottom row), also follow patterns seen in previous multimodel comparisons (Allen & Ingram, 2002; Knutti & Sedláček, 2012; Samset et al., 2016). We find a general increase of 2–6% per °C, dominated by the tropical ocean regions and a shift of the Intertropical Convergence Zone although with significant intermodel differences. Removing aerosol emissions broadly reinforces this pattern. Natural variability in precipitation reduces the model agreement, as discussed elsewhere (see, e.g., Deser et al., 2012, and Schaller et al., 2011). For regions where the response to GHG and aerosol changes are both significant, however, the ASR is again seen to be well above unity in the Northern Hemisphere, indicating a higher regional hydrological sensitivity to aerosol removal than to GHG dominated warming.

We have here found that the global, annual, multimodel mean temperature and precipitation response to fully removing anthropogenic aerosols is half to a third of that of a GHG-driven warming of around 1.5°C. Extreme weather indices may not simply scale with global surface temperature, due to their dependence on local physical conditions and regional forcing patterns (Boucher et al., 2013; Sillmann, Kharin, Zwiers, et al., 2013; Westra et al., 2014). In the following, we compare the regional sensitivities of TXx, RX5D, and CDD changes due to GHG-dominated warming, with removal of anthropogenic aerosol emissions.

**Figure 3.**
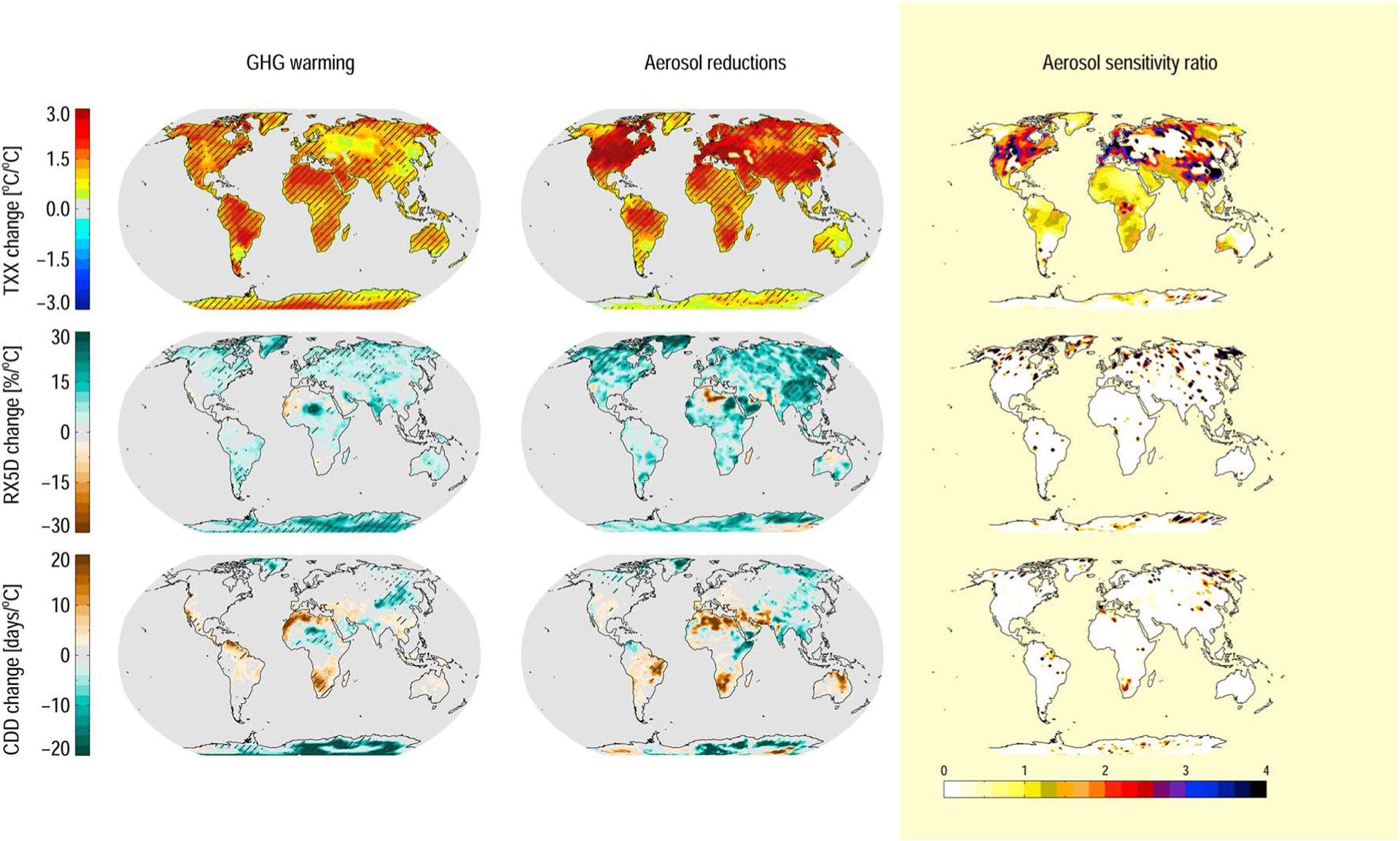
Extreme weather sensitivity to changes in (top row) long-lived GHG and (middle row) aerosol removal. Mean of four models. (bottom row) Aerosol response pattern (ASR). The hatching shows where all four models agree on the sign of the change. TXx: maximum daily temperature, annual mean. RX5D: maximum 5 day precipitation. CDD: consecutive dry days. ASR is only plotted where models agree on the sign of the change for both the GHG and aerosol simulations.

Figure 3 shows multimodel mean maps of the changes to the extreme weather indices, per degree of global mean surface warming, and their aerosol sensitivity ratios, computed as for Figure 2. Looking first at the GHG-dominated warming (Figure 3, left column), we find a high sensitivity of the maximum annual daily temperature (TXx; Figure 3, top row) for all land areas. In Northern Africa, the Middle East, and South America the maximum annual daily temperature increases by up to 2°C per degree of warming. All models agree on the sign of the change over most of the land area (hatching). Maximum 5 day precipitation (RX5D; Figure 3, middle row) increases with global warming for much of the global land area. As expected for precipitation-related indices, model agreement is low. In particular, we note that agreement is poor in the aerosol source regions of Europe, East Asia, and the U.S. For consecutive dry days (CDDs; Figure 3, bottom row), the models predict an increase with warming in parts of Africa, and a strong decrease of up to 10 days per °C in regions of the inner Eurasian continent, but again with poor agreement between the models.

We note here that the patterns seen for the change in extreme weather indices broadly follow those found in a recent study on the climate impacts of 1.5°C and 2.0°C warming (Schleussner et al., 2016). However, that study used a 1986–2005 reference period and looked at the 1.5°C warming period in the CMIP5 ensemble (occurring around 2030–2040 for RCP8.5), where the scenarios already include some aerosol emission reduction.

The sensitivities of extreme weather to removing anthropogenic aerosol emissions (Figure 3, middle column) show similar patterns to those from GHG-dominated warming. This is expected, as much of the change is likely associated with the mean warming of the climate system stemming mainly from the removal of sulfate cooling. As for temperature and precipitation change, however, we find higher sensitivities in the Northern Hemisphere. A main reason for this is likely a higher surface temperature change locally where aerosols are removed. Regionally, we note an elevated sensitivity of TXx (ASR > 3) over the U.S., Europe, and East Asia. RX5D sensitivities are stronger for aerosol reductions than for GHG removal, with higher consistency between models. For CDD, the model agreement is low, but we can conclude that the patterns appear similar for both cases. Consequently, the ASR calculation tells little for the RX5D and CDD changes, although it is >1 for the few locations in the Northern Hemisphere where we can make an assessment.

**Figure 4.**
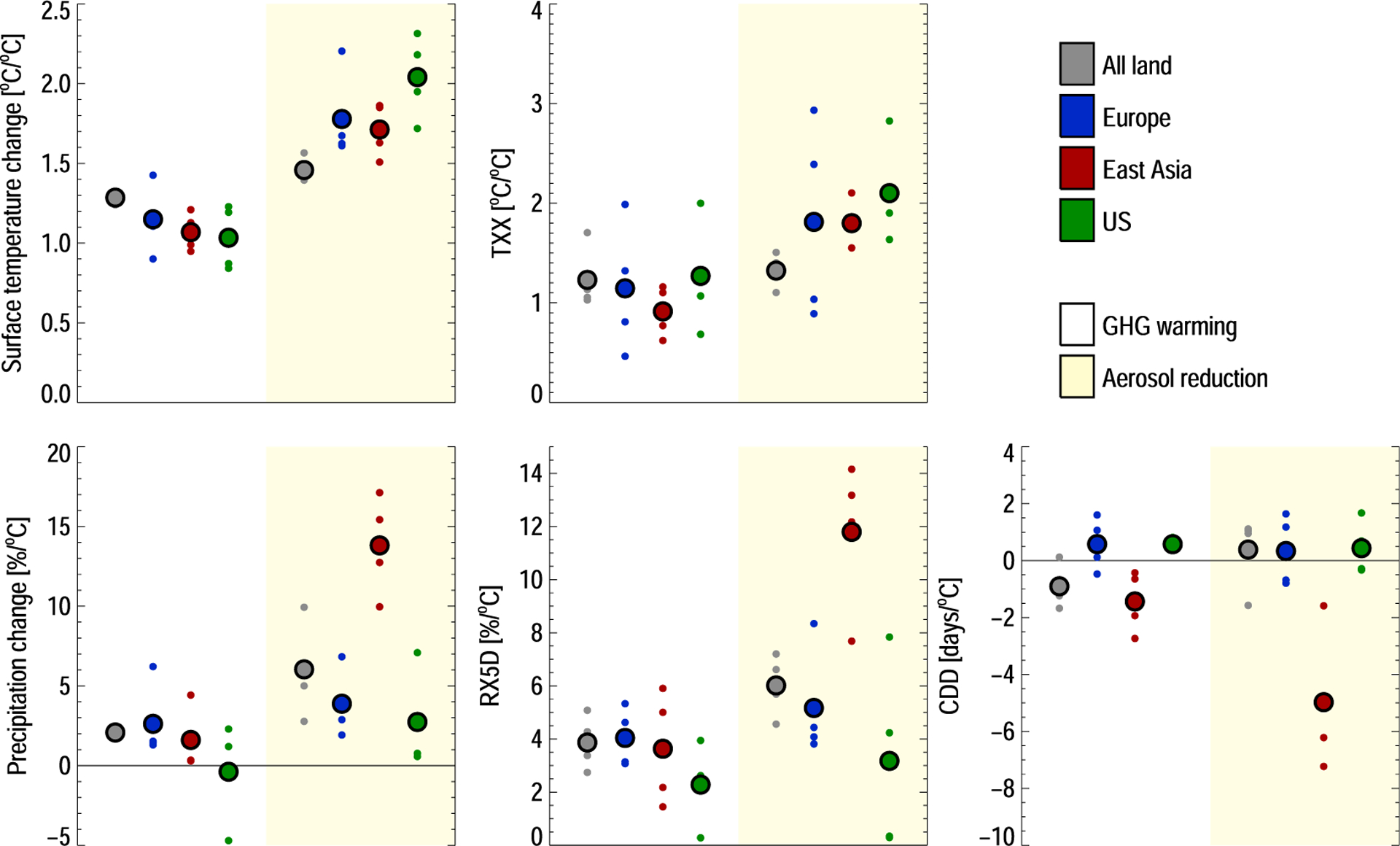
Land area mean changes to temperature, precipitation, and extreme weather indices, per degree of global mean surface temperature change. Globally (grey) and for three major aerosol emission regions (colors). The large circles show multimodel means; the small circles show individual model values. The left values are for GHG-induced warming; the right values (on yellow background) are for aerosol emission reductions.

In Figure 4, we aggregate the changes seen in Figure 3, globally and for three selected aerosol emission regions (Europe, East Asia, and the U.S.). For GHG warming, the temperature change over land (Figure 4, top left) is 30% stronger than the global mean, with little intermodel variation. For the aerosol emission regions, the land-to-global response ratio for GHG warming is consistent with unity. Aerosol emission reductions, however, show a 50% stronger land temperature increase than globally, consistent with previous findings (Shindell, 2014). The major aerosol emission regions see even higher ratios under aerosol removal, due to strong reductions in local sulfate induced cooling. Similar conclusions hold for precipitation (Figure 4, bottom left), except for a much stronger response to aerosol reductions in the East Asia region (15% per °C).

For climate extremes, TXx and RX5D follow the patterns of temperature and precipitation change, respectively. For all three major aerosol source regions, we find that the hottest day temperatures and maximum precipitation will change more for heating due to aerosol emission reductions than for a similar global warming due to greenhouse gases. This difference needs to be kept in mind when discussing the potential regional implications of a given combination of GHG mitigation and air quality measures. A further implication is that even if global warming is kept below 1.5 or 2°C, regional climate may change as the composition of climate forcers changes over time.

For CDD, the models predict changes that differ in sign. One major exception is, again, East Asia, where the strong increase in precipitation also induces a reduction in the number of dry days in all models.

Multiple processes likely underlie the differences between GHG and aerosol perturbations shown in Figure 4. However, we expect differences in land area heating to be dominant factor. Figure S6 shows the regional responses normalized by local temperature change rather than global. Here we find more similar response patterns for mean precipitation and extreme indices (mean temperature is identical by construction), indicating that the main difference has a thermodynamic origin. The one major deviation is East Asia, where the precipitation responses are still significantly stronger for aerosol reductions. We attribute this to removal of rapid adjustments from black carbon emissions, which are known to reduce regional precipitation (Samset et al., 2016).

## 4. Discussion and Conclusions

We have shown how climate impacts from the expected strong reductions in anthropogenic emissions of BC, organic carbon, and SO_2_ may compound with those from GHG-driven global warming. The models simulate an additional global warming of around 0.7°C when fully removing anthropogenic aerosols, with a model range of (0.5, 1.1)°C. This is comparable in magnitude to the 1°C already realized since preindustrial times.

Populated regions see stronger changes in temperature, precipitation, and extremes than the land area mean. In the major aerosol source regions, both temperature, precipitation, and extreme weather indices are more sensitive to a removal of anthropogenic aerosols than to GHG increases, per degree of global mean temperature change. The geographical pattern of changes to the extreme weather indices is also different from that expected from a similar GHG-driven surface temperature increase, with elevated sensitivities to aerosol changes in the Northern Hemisphere. We highlight East Asia as a region where extreme precipitation is particularly sensitive to a reduction in aerosol emissions.

One clear limitation of the present study is the low number of models, compared to larger multimodel inter-comparisons such as CMIP5. We note, however, that the host models used here span much of the range of previously reported climate sensitivities (Forster et al., 2013) and precipitation responses to identical perturbations (Samset et al., 2016). A related issue is the known diversity in precipitation responses in present climate models (Knutti & Sedláček, 2012), which also affects extreme weather index calculations. The spread in calculated impacts shown here should therefore be taken as indicative, rather than an upper bound. A further question, not unique to the present study, is the ability of the current generation of global models to simulate climate extremes. We note that the performance of the CMIP5 models has recently been evaluated in this context (Fischer et al., 2013; Sillmann, Kharin, Zhang, et al., 2013), with results indicating reasonable model skills when aggregated over larger regions, such as used in the present study.

We note that the range of GHG warming simulated here, for present-day aerosols, ((1.1, 1.7)°C), occurred for a CO_2_ concentration range of (406, 430) ppm. For example, the GISS model found a centennial-scale global warming of 1.4°C for only 406 ppm, a level which is close to the observed present-day values. The differences in sensitivity shown here highlight the need for studies of future climate change to carefully consider not just their net forcing and resulting global mean surface temperature but also the detailed balance between greenhouse gases and aerosols in emission pathways. The differences in spatial pattern between climate forcing and response due to greenhouse gases and aerosols mean that for low global warming scenarios, e.g., consistent with the 1.5° target, the realized climate impacts will depend significantly on the path we take toward a global temperature goal.

## Supplementary Material

Supp1

## Figures and Tables

**Table 1:** Absolute Model Responses to GHG-Dominated Warming (Difference Between Simulations ii and i; see section 2) and Aerosol Reduction (Difference Between Simulations iii and ii)

	Greenhouse gas-dominated warming					Aerosol reduction				
	Δ*T* (°C)	Δ*P* (%)	ΔTXX (°C)	ΔRX5D (%)	ΔCDD (days)	Δ*T* (°C)	Δ*P* (%)	ΔTXX (°C)	ΔRX5D (%)	ΔCDD (days)
CESM1	1.7	3.1	1.8	5.5	−1.5	0.5	1.5	0.6	3.1	0.5
GISS	1.4	2.2	1.4	3.7	−2.3	0.5	1.8	0.7	3.4	0.5
HadGEM2	1.2	2.1	1.2	5.0	0.1	1.1	2.6	1.5	4.7	−1.7
NorESM1	1.1	0.9	1.5	3.8	−0.9	0.6	3.1	0.9	5.3	−1.2

*Note*. Δ*T* and Δ*P* are global means, while ΔTXX, ΔRX5D, and ΔCDD are land surface means.

## Key Points:

- Aerosol emission removal can warm the climate by more than 0.5°C
- Key climate variables are more sensitive to aerosol removal than to GHG increase
- Regional impacts of 1.5°C warming depend on the balance between aerosol and GHG forcing
