# Supplementary material for: Climate Impacts From a Removal of Anthropogenic Aerosol Emissions
Source: Geophys Res Lett. Author manuscript; Available in PMC 2020 Aug 14. (PMC7427631; doi:10.1002/2017GL076079)
Supplement: Supp1 [file NIHMS980486-supplement-Supp1.docx]

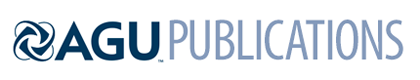


*Geophysical Research Letters*

Supporting Information for

**Climate impacts from a removal of anthropogenic aerosol emissions**

B. H. Samset^1^, M. Sand^1^, C. J. Smith^2^, S. E. Bauer^3^, P. M. Forster^2^, J. S. Fuglestvedt^1^, S. Osprey^4^, C.-F. Schleussner^5^

1: CICERO Center for International Climate and Environmental Research – Oslo, Norway
2: University of Leeds, Leeds, United Kingdom
3: NASA Goddard Institute for Space Studies and Columbia Earth Institute, New York, NY, USA
4: National Centre for Atmospheric Science and Department of Physics, University of Oxford, Oxford, United Kingdom
5: Climate Analytics, Berlin, Germany

**Contents of this file**

Figures S1 to S6.

**Introduction**

Figure S1 shows the year-to-year variability of global, annual mean temperature for the four participating models, and the full evolution of the precipitation and top-of-atmosphere energy imbalance for one model.

Figures S2-S5 show the geographical responses to a greenhouse gas increase and an aerosol reduction, for the individual models used in our study. The maps show the average of simulated years 51-100 after the perturbations. “GHG warming” refers to the difference between a GHG increase above present day conditions and a preindustrial baseline (simulation **ii** minus **i**, as defined in the Methods section), which “Aerosol reduction” refers to a further removal of all anthropogenic emissions of sulfate, black carbon and organic carbon (simulation **iii** minus **ii**).

Figure S6 repeats Figure 4 of the main paper, but normalized to regional rather than global mean temperature change.

**
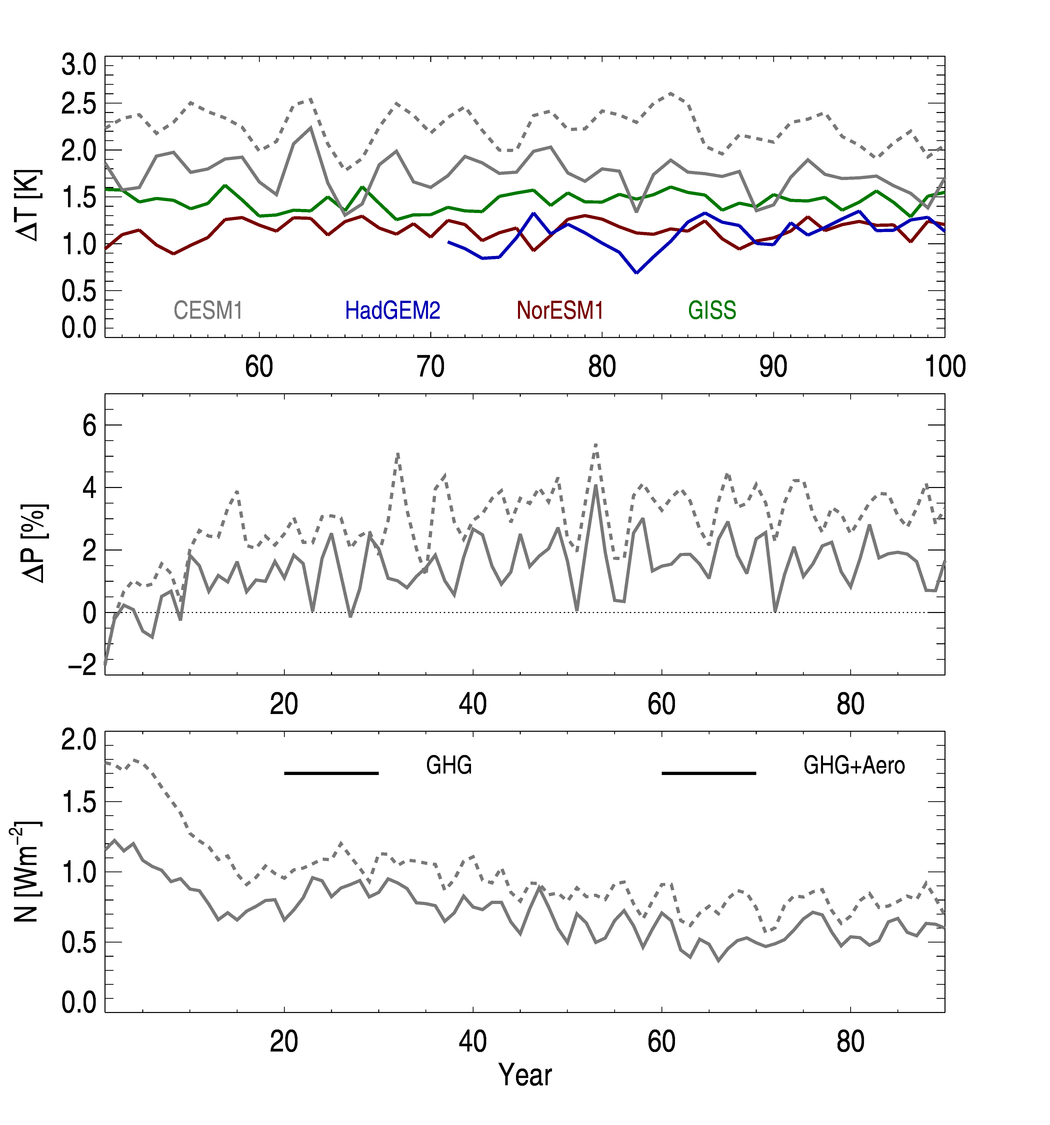
**

Figure S1: Documentation of model stability. The top panel shows the year-to-year variability of global, annual mean temperature change for the four participating models, over the period used for the present analysis. The middle and bottom panels show the full evolution of global, annual mean precipitation change and top-of-atmosphere energy imbalance for one model (CESM1). See main text for discussion of long-term model equilibration.


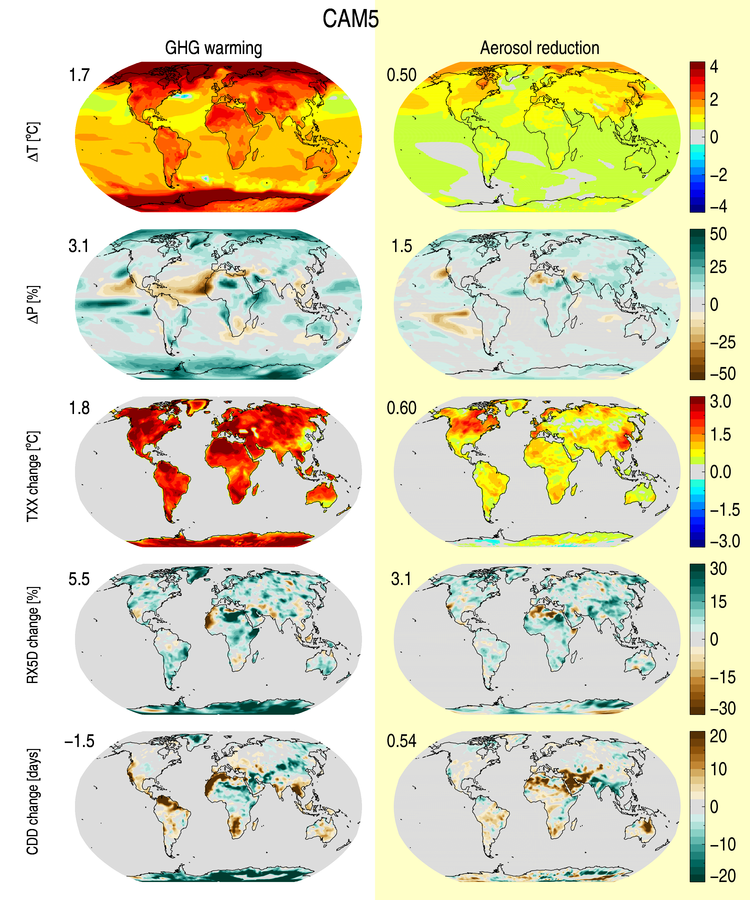


Figure S2: CESM CAM5 responses to GHG-dominated warming (historical + additional CO_2_, left) and anthropogenic aerosol removal (right). Numbers show global means for ΔT and ΔP, and land surface means for TXX, RX5D and CDD changes.


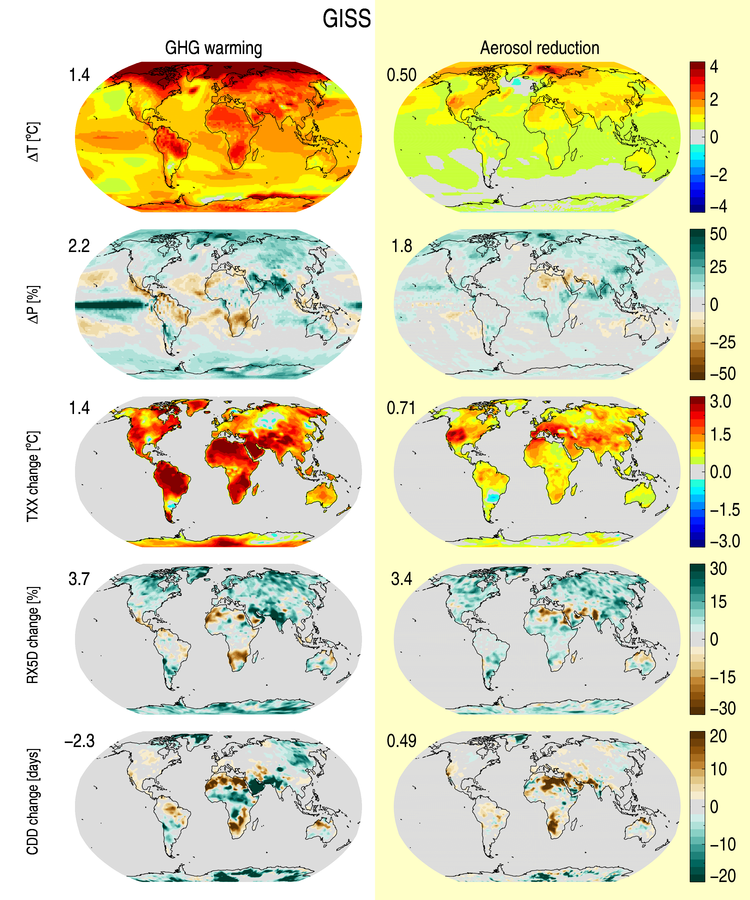


Figure S3: As S2, for GISS-E2-R OMA.


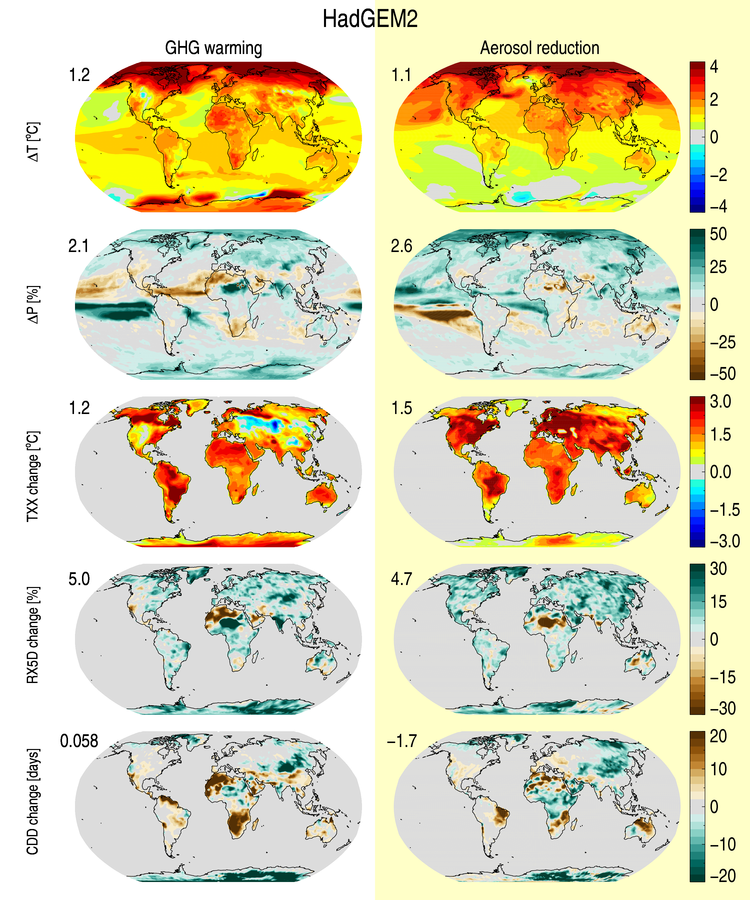


Figure S4: As S2, for HadGEM2-CCS.


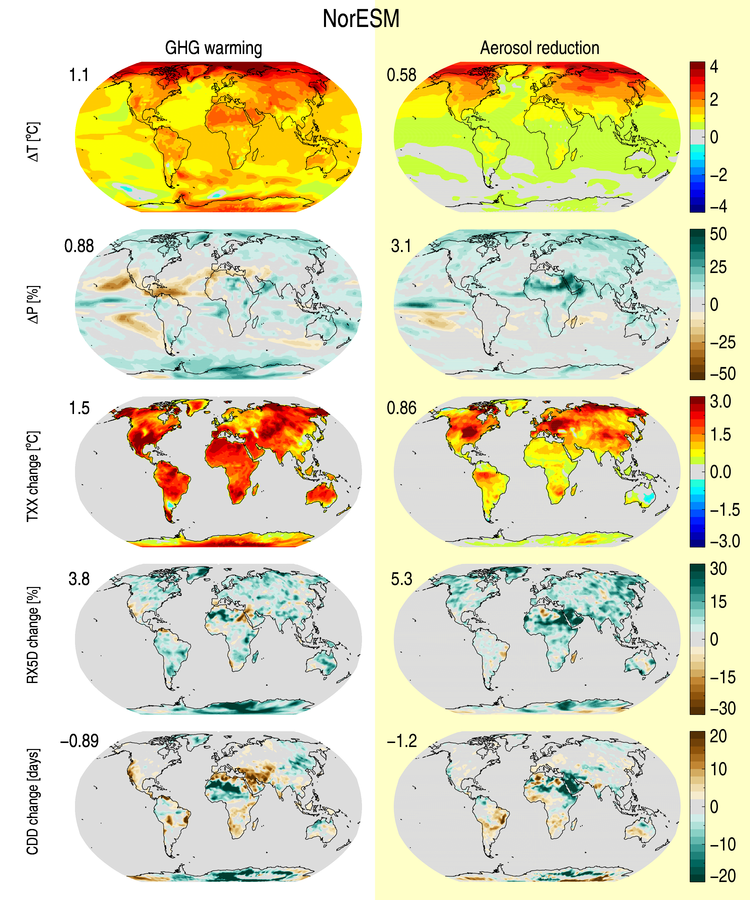


Figure S5: As S2, for NorESM1.


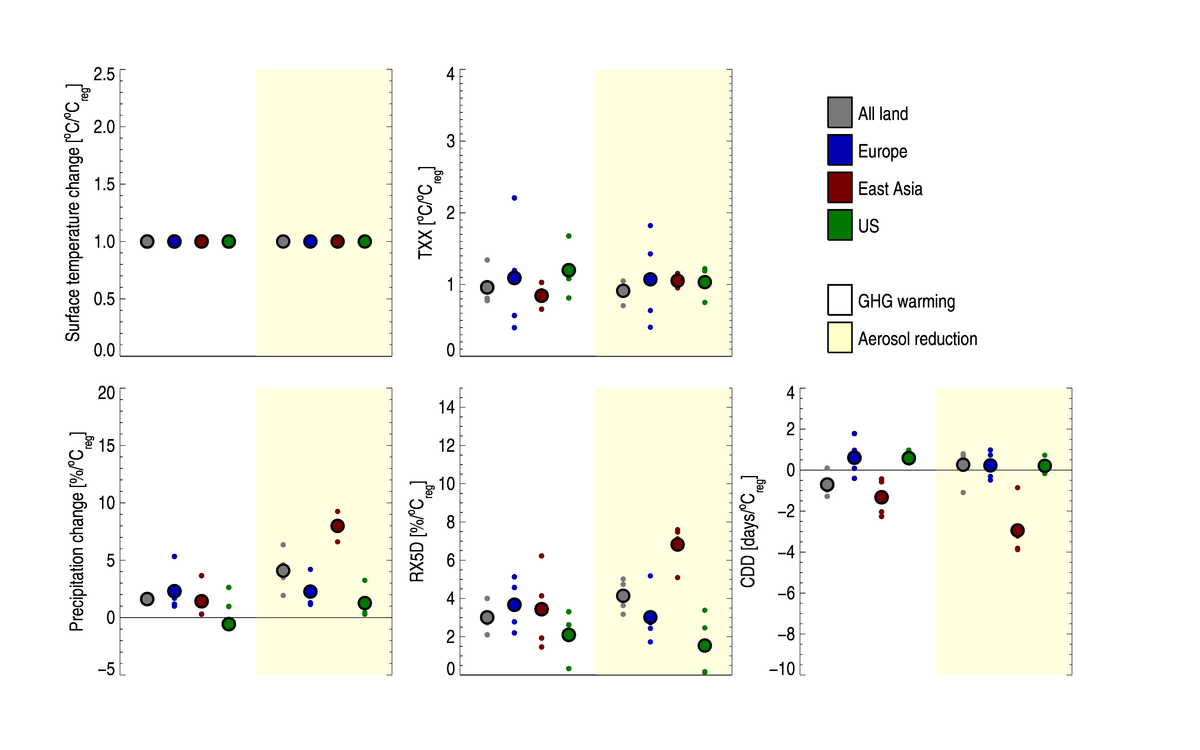


Figure S6: Land area mean changes to temperature, precipitation and extreme weather indices, per degree of mean surface temperature change within the region. (As Figure 4 in the main paper, but normalized to regional temperature change rather than global mean.) Large circles show multi-model means, small circles show individual model values. The left values are for GHG-induced warming, the right values (on yellow background) are for aerosol emission reductions.
